# The Relief of Unilateral Painful Thoracic Radiculopathy without Headache from Remote Spontaneous Spinal Cerebrospinal Fluid Leak

**DOI:** 10.1155/2016/4798465

**Published:** 2016-04-17

**Authors:** Byung-chul Son, Sang-woo Ha, Si-hoon Lee, Jin-gyu Choi

**Affiliations:** ^1^Department of Neurosurgery, Seoul St. Mary's Hospital, College of Medicine, The Catholic University of Korea, Seoul 06591, Republic of Korea; ^2^Catholic Neuroscience Institute, College of Medicine, The Catholic University of Korea, Seoul 06591, Republic of Korea; ^3^Department of Neurosurgery, Chosun University Hospital, Chosun University, Gwangju, Republic of Korea

## Abstract

Spontaneous intracranial hypotension (SIH) caused by spontaneous spinal cerebrospinal fluid (CSF) leaks produces orthostatic headaches. Although upper arm pain or paresthesia is reportedly associated with SIH from spontaneous spinal CSF leak in the presence of orthostatic headache, low thoracic radicular pain due to spontaneous spinal CSF leak unassociated with postural headache is extremely rare. We report a 67-year-old female who presented with chronic, positional radicular right T11 pain. Computed tomography myelography showed a spontaneous lumbar spinal CSF leak at L2-3 and repeated lumbar epidural blood patches significantly alleviated chronic, positional, and lower thoracic radiculopathic pain. The authors speculate that a chronic spontaneous spinal CSF leak not severe enough to cause typical orthostatic headache or epidural CSF collection may cause local symptoms such as irritation of a remote nerve root. There might be considerable variabilities in the clinical features of SIH which can present a diagnostic challenge.

## 1. Introduction

Most spinal cerebrospinal fluid (CSF) leaks are iatrogenic, occurring after lumbar puncture, epidural injection, or spinal surgery. Spontaneous intracranial hypotension (SIH) is a postural headache syndrome unrelated to dural puncture and it is an uncommon but increasingly recognized clinical syndrome associated with reduced CSF pressure or CSF volume caused by spontaneous CSF leaks [1–5]. The incidence has been estimated at 5 per 100,000 per year, with a peak around 40 years of age [5]. SIH affects women more frequently than men, with a female-male ratio of approximately 2 : 1 [4, 5]. Spontaneous spinal CSF leaks are the typical cause of SIH [5, 6]. Such spontaneous CSF leaks may result from a simple dural rent or fragile arachnoid cysts, often in the presence of an underlying but previously unrecognized generalized connective tissue disorder [5–8]. An orthostatic headache is the prototypical manifestation, but other headache patterns occur as well, and associated symptoms are common [5].

When onset of such headache is spontaneous, the diagnostic challenge is created even though SIH has been recognized for more than 6 decades and the diagnosis has been greatly aided by magnetic resonance imaging (MRI) [4]. Typical brain MRI findings include subdural fluid collections, pachymeningeal enhancement, engorgement of venous structures, pituitary hyperemia, and downward displacement of the brain [4–11]. Because spinal CSF leaks generally do not cause any local symptoms [6], they frequently remain undetected unless actively looked for in a patient suspected of SIH [4, 5]. The precise cause of spontaneous spinal CSF leaks remains largely unknown, but an underlying structural weakness of the spinal meninges generally is suspected [6].

Until now, the symptoms and signs of SIH from spontaneous spinal CSF leak are reported to be associated with the head and the upper extremity [4, 9]. A thoracolumbar origin is extremely rare. Although a case of cervical radiculopathy causing weakness of shoulder elevation due to engorgement of the cervical epidural vein [9] and a case of paresthesia in the upper extremity [4] have been reportedly associated with SIH, an isolated presentation of unilateral low thoracic radiculopathic pain from spontaneous CSF leak without typical positional headache has not been reported. We report a case of spontaneous lumbar CSF leak presenting as a chronic, intractable, positional, unilateral lower thoracic radiculopathic pain, not associated with the typical symptom of orthostatic headache.

## 2. Case Presentation

A 67-year-old female presented with a 12-month history of chronic radicular pain along the course of right T11. The pain was described as mainly aching, with extreme severity (7-8/10 on the numeral rating scale (NRS) ranging from 0 to 10). It was aggravated with position change from lying to sitting, and daily activity and walking did not influence the pain. There was no precipitating event before the onset of right thoracic pain and its onset was insidious. The pain was not relieved with nonsteroidal anti-inflammatory drugs, tramadol (300 mg/day), and 20 mg oxycodone b.i.d. and was so agonizing that treatment with repeated injections of diclofenac sodium and on a regular schedule of epidural blocks, intercostal blocks of T11, and tender point injections was done two times a week for a year. However, repeated blocks were unsuccessful. She underwent right-sided L4-5 hemilaminectomy because the pain radiated to the lateral flank and was misdiagnosed as lumbar stenosis 5 months after pain onset. Hemilaminectomy was not effective. Finally, she was referred to our outpatient clinic under the diagnosis of failed back surgery syndrome.

On examination, the pain was typically present along the course of the right T11 dermatome from the lower trunk radiating to the right lower abdomen. There was no sensory disturbance including right T11 dermatome and right leg, and no allodynia, tenderness, or limitation of motion of low back and trunk was observed. Laboratory examinations were normal including the erythrocyte sedimentation rate, C-reactive protein, and blood sugar level (95 mg/dL). A Congo red stain of the abdominal fat to rule out amyloidosis was negative. Plain X-rays of lower thoracic and lumbar spine were unremarkable and no pathologic lesion to explain right T11 pain was found in the MRI of the lower thoracic and lumbar spine. Electromyography including nerve conduction study revealed no evidence of radiculopathy or peripheral neuropathy.

**Figure 1:**
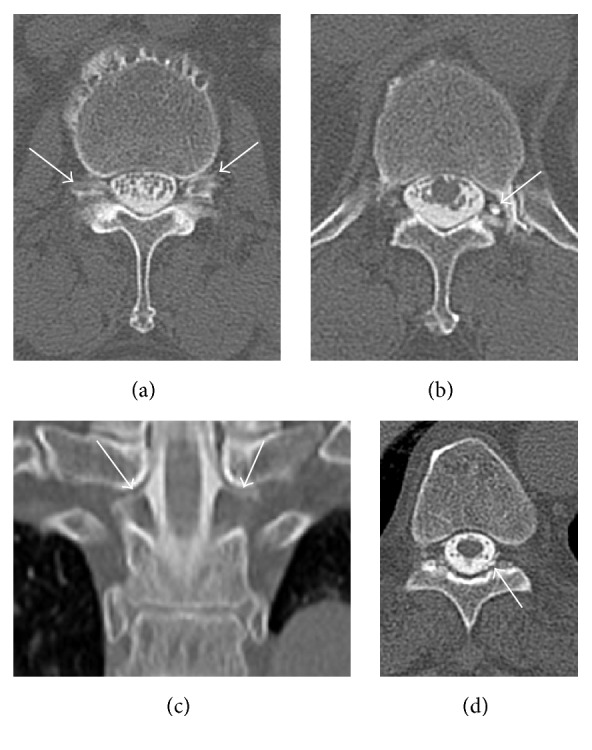
Computed tomography (CT) myelograms showing spontaneous spinal cerebrospinal fluid (CSF) leaks. (a) Bilateral CSF leaks (*arrows*) at L2-3 levels. (b) A contrast-filling nerve root sheath (*arrows*) at left T12-L1 level. (c) A coronal reconstruction image showing bilateral contrast-filling nerve root sheaths (*arrows*) at T1 level. (d) A small amount of contrast leak (*arrow*) in left T10 epidural space.

As an evaluation for failed back surgery syndrome, myelography computed tomography (CT) was performed. There was no lesion found causing right T11 pain. However, a prominent CSF leak was noted bilaterally at the level of L2-3 (Figure 1(a)) and contrast-filling nerve root sheath was identified at left T12 (Figure 1(b)), left L1 (Figure 1(c)), and bilateral T1. A small amount of epidural contrast leakage was found at the T10 level (Figure 1(d)). There was no pseudomeningocele or CSF leak at the laminectomized L4-5 level. Under suspicion of spontaneous spinal CSF leak, lumbar CSF examination was done 2 days later with adequate hydration. The CSF opening pressure was 8 cmH_2_O in the recumbent position. The CSF examination showed 10 cells/mm^3^ and a protein level of 35 mg/dL. No erythrocytes were found.

On the basis of the CT myelographic finding of a CSF leak, the provisional diagnosis was spontaneous spinal CSF leak causing low thoracic radiculopathic pain. Considering the chronicity and medical intractability of the radicular pain, combined with the radiological finding of spontaneous CSF leaks, an epidural blood patch (EBP) with 15 mL of autologous blood was performed at the L1-2 level. Immediately after the blood patch, she reported about 30% relief of T11 radicular pain (4–6 on NRS-11). A repeat lumbar EBP was performed one week later, which achieved an additional 30% pain relief (2–5 on NRS-11), and a total of 60% pain relief over baseline was achieved. At 2 weeks after the blood patch, myelographic CT and enhanced lumbar spine MRI were performed to assess the degree of spinal CSF leaks. There was no identifiable CSF leak at the L2-3 level. However, contrast-filling in bilateral nerve root sheaths without CSF leak was identified at T12-L1, L2-3, and L3-4 levels. The small amount of epidural contrast leakage at the level of T10 had disappeared.

In the repeated enhanced lumbar spine MRI, there was no typical finding of spontaneous CSF leak, such as epidural CSF collection. However, subtle epidural enhancement was noted from T10 to L3, and a remnant of the injected epidural blood patch was observed. After achieving about 60% pain relief, she was discharged and was maintained with medication (10 mg oxycodone b.i.d., 400 mg gabapentin t.i.d., 50 mg tramadol/acetaminophen t.i.d., and 10 mg amitriptyline hs/a day).

A repeated myelographic CT was performed 3 months after the first EBP showed the same findings obtained in the second myelographic CT, bilateral contrast-filling nerve root sheaths without CSF leak evident at the T12-L1, L2-3, and L3-4, L4-5 levels. An additional blood patch was not performed as the patient could tolerate low thoracic pain (NRS 2–4/10). Thus, the pain in this patient which was of 12 months' duration and severe was relieved after two epidural blood patches to a mild to moderate level (2–4 on NRS-11) at 12-month follow-up.

## 3. Discussion

### 3.1. Spontaneous Intracranial Hypotension (SIH) and Spontaneous CSF Leaks

Intracranial hypotension (IH) is a clinical syndrome in which absolute or relative hypovolemia of CSF results in various clinical symptoms [2]. Causes of IH can be either spontaneous or secondary to lumbar puncture, spine surgery, and chiropractic manipulation [1, 3, 9, 12, 13]. Many patients with IH present without known predisposing factors or a major trauma, and the condition has been called “spontaneous” IH (SIH). SIH is caused by spontaneous CSF leaks and is characterized by orthostatic headache (i.e., headache aggravated with standing up and relieved by lying down). Associated neurologic symptoms include nausea, vomiting, dizziness, photophobia, cranial nerve palsies such as diplopia, tinnitus, hearing disturbance, stiffness, and back pain, and radicular symptoms, such as pain or paresthesia of the upper extremity [4, 5, 9]. True radicular symptoms, such as pain and paresthesia, are extremely rare.

Despite recent awareness of SIH by physicians, the diagnosis of SIH can be missed initially and the diagnostic delay is significant [4]. In a series of 18 consecutive series of SIH [4], 17 (94%) were initially given incorrect diagnoses, including migraine, meningitis, subarachnoid hemorrhage, cervical strain, and cervical radiculopathy [4]. This exposes patients to risks associated with treatment for the disorders that mimic SIH; the importance of increasing awareness of SIH causing headache has been stressed [4, 11].

### 3.2. Differential Diagnosis

As a differential diagnosis of isolated T11 radiculopathic pain, both structural lesions and nonstructural peripheral neuropathic pain should be taken into account. For structural lesions such as foraminal stenosis or disc pathology, there was no obvious pathologic lesion possibly abutting the right T11 root on both MRI and myelographic CT scans. A radionuclide bone scintigraphy was negative and a neoplasm or benign tumor involving peripheral nerve root was also excluded with an enhanced MRI study. Diverse peripheral neuropathic lesion or systemic metabolic disease could involve a thoracic nerve root. Most commonly, diabetic neuropathy, nonvesicular form of herpes zoster (zoster sine herpete, ZSH), or sarcoid infiltration of nerve root could be considered.

A syndrome of painful unilateral or symmetrical multiple neuropathy tends to occur in older patients with relatively mild or even unrecognized diabetes [14]. However, the patient did not show a history of diabetes and blood sugar level was normal. Diabetic amyotrophy typically involves lumbar roots and can cause severe aching pain with superimposed lancinating jabs.

Although zoster sine herpete (ZSH) could manifest as chronic nonvesicular thoracic radiculopathy [15, 16], this patient did not show hyperaesthesia or allodynia to light touch which are the characteristics of ZSH. There was no granulomatous lesion in the chest, skin, and the eye which could be suspected for sarcoid infiltration and the test for systemic amyloidosis was negative also. Other possibilities of radiating pain caused by intra-abdominal or retroperitoneal pathology were ruled out by an abdominal CT scan during evaluation. In this case, we believe radicular pain occurred because of spontaneous CSF leak without headache.

### 3.3. Cause of Right T11 Pain from Spontaneous CSF Leak

Spontaneous CSF leak as a cause of the right T11 radicular pain in the present case may be questioned because there has been no reported case of SIH causing lower thoracic radiculopathy. Furthermore, there was no orthostatic headache. No typical enhanced MRI of the brain was able to be obtained and CSF pressure was normal. The only positive finding that supports the diagnosis of SIH due to spontaneous CSF leak is CT myelographic evidence of CSF leak in the thoracolumbar junction area and subsequent improvement of radicular pain after EBPs.

However, considerable variability of the clinical features of SIH has been reported [4–6, 8]. Although an orthostatic, positional headache is a typical symptom of spontaneous CSF leak, absence of orthostatic headache in spite of typical meningeal enhancement on brain MRI and low CSF pressure has been reported. Mokri et al. [17] reported three patients—two with overdraining CSF shunts and one with proven CSF leak—with typical pachymeningeal enhancement but without headache. Hochman and Naidich [18] also reported two patients with overdraining, long-standing ventricular shunts without any headache despite typical diffuse meningeal enhancement and documented low CSF pressure on lumbar puncture. Some patients deny having any headache, usually when other symptoms of SIH, such as posterior neck pain, nausea, and photophobia, predominate the clinical picture [5]. Regarding typical MRI finding of pachymeningeal enhancement, up to 20% of patients with SIH never develop enhancement, or any of the other abnormalities on brain MRI [13, 19, 20]. Unfortunately, we could not examine the brain MRI in the present case, as the patient refused to have this test as her complaint was only low thoracic pain. It is known that CSF opening pressure in SIH is typically less than 60 mmH_2_O (reference range, 65–195 mmH_2_O), which can be unmeasurable or even negative [21]. However, some patients with SIH have consistently normal CSF pressure [5, 6].

Indeed, the CT myelographic findings of spontaneous CSF leak in the current case were an unexpected finding during evaluation of right T11 radicular pain. Spontaneous CSF leak was most prominent at L2-3 and there was a scant amount of epidural contrast leak at T10. The CSF leaks at L2-3 and T10 disappeared after EBP with concomitant pain relief. In addition, multiple areas of contrast-filling nerve root sheaths were observed on the left side of T12-L1, left L1, and bilateral T1 in initial examination. However, the areas with contrast-filling nerve root sheaths were found bilaterally at the level of T12-L1, L2-3, and L3-4 in the second examination and bilaterally in T12-L1, L2-3, L3-4, and L4-5 in the follow-up examination at 3 months. The exact significance of contrast-filling along the course of the nerve root sheath is uncertain. We think there is a possibility that these changes may implicate an underlying structural weakness of spinal meninges, which causes spontaneous spinal CSF leak [6, 9], and sealing of spinal CSF leak might induce some redistribution of intraspinal CSF pressure. In this patient, contrast-filling was prominent in the left T12-L1 and this had become prominent in the right T12-L1 in the follow-up myelographic CT.

Relatively less attention is typically given to spinal MRI in the diagnosis of SIH, mainly because it is not particularly effective in localizing the CSF leak [5]. However, many spinal MRI manifestations of SIH have now been described, such as dilated epidural or intradural veins, dural enhancement, meningeal diverticula, epidural CSF collections, and collapse of the dural sac [10, 12, 18, 22–25]. In this case, the initial spinal MRI was not informative because there was no clinical suspicion of spontaneous spinal CSF leak. MRI did not reveal characteristic signs of spinal CSF leak, such as epidural CSF collection or pseudomeningocele, or a dilated epidural vein causing compression of low thoracic nerve root. Enhancement was not performed. The enhanced spine MRI taken 2 weeks after the initial EBP again showed no typical findings of spontaneous CSF leak. However, on enhancement study in this occasion, subtle leptomeningeal enhancement was found from the T12 to L3 level.

It seems that the severity of the spontaneous CSF leak in the present case was obviously milder than those reported in the literature. This CSF leak was not severe enough to cause an orthostatic headache and typical spinal MRI findings of a CSF collection epidurally and in paraspinal muscles [3, 22, 23]. However, there were definite spontaneous CSF leaks at the L2-3 level and a chronic, low thoracic radiculopathic pain of 12-month duration, which showed a positional pain and which responded to L1-2 epidural blood patch. We speculate that a less severe type of spontaneous spinal CSF leak might cause intracranial hypotension and subsequent stretching or irritation of a lower thoracic root which would result in chronic radicular pain.

Assuming that thoracic radicular pain unassociated with anatomical pathology presents without positional headache, clinical suspicion of spontaneous CSF leak would not be possible in daily clinical practice if spinal MRI does not show an extensive epidural fluid collection or paraspinal CSF extension. As shown in present case, spontaneous spinal CSF leak as a cause of unexplained, chronic thoracic radicular pain inevitably requires a process of diagnosis of exclusion. We suggest that if unexplained thoracic radicular pain presents without orthostatic headache, even without the brain and spinal findings of SIH, spontaneous spinal CSF leak should be included in the list of differential diagnosis.

## 4. Conclusion

The occurrence of cervical unilateral radiculopathic pain caused by spontaneous spinal CSF leak has been reported in association with a typical orthostatic headache. The development of an isolated chronic lower unilateral thoracic radicular pain without orthostatic headache may be a rare manifestation of a remotely located spontaneous CSF leak. As shown in our case, disabling lower thoracic unilateral radicular pain of 12-month duration from an L2-3 CSF leak responded at 12-month follow-up after two epidural blood patches. We speculate that a chronic, milder pattern of spontaneous spinal CSF leak may cause a radicular pain without typical symptoms of intracranial hypotension. Spontaneous spinal CSF leak may have a different presentation of remote radicular pain and presents a variability to the clinical features of spontaneous intracranial hypotension.

## Acronyms

CSF: Cerebrospinal fluid
EBP: Epidural blood patch
Myelographic CT: Myelography computed tomography
SIH: Spontaneous intracranial hypotension.

## References

[1] Miyazawa K., Shiga Y., Hasegawa T. (2003). CSF hypovolemia vs. intracranial hypotension in ‘spontaneous intracranial hypotension syndrome’. *Neurology*.

[2] Mokri B. (1999). Spontaneous cerebrospinal fluid leaks: from intracranial hypotension to cerebrospinal fluid hypovolemia—evolution of a concept. *Mayo Clinic Proceedings*.

[3] Paldino M., Mogilner A. Y., Tenner M. S. (2003). Intracranial hypotension syndrome: a comprehensive review. *Neurosurgical Focus*.

[4] Schievink W. I. (2003). Misdiagnosis of spontaneous intracranial hypotension. *Archives of Neurology*.

[5] Schievink W. I. (2006). Spontaneous spinal cerebrospinal fluid leaks and intracranial hypotension. *The Journal of the American Medical Association*.

[6] Schievink W. I., Meyer F. B., Atkinson J. L. D., Mokri B. (1996). Spontaneous spinal cerebrospinal fluid leaks and intracranial hypotension. *Journal of Neurosurgery*.

[7] Schievink W. I., Torres V. E. (1997). Spinal meningeal diverticula in autosomal dominant polycystic kidney disease. *The Lancet*.

[8] Schrijver I., Schievink W. I., Godfrey M., Meyer F. B., Francke U. (2002). Spontaneous spinal cerebrospinal fluid leaks and minor skeletal features of Marfan syndrome: a microfibrillopathy. *Journal of Neurosurgery*.

[9] Albayram S., Wasserman B. A., Yousem D. M., Wityk R. (2002). Intracranial hypotension as a cause of radiculopathy from cervical epidural venous engorgement: case report. *American Journal of Neuroradiology*.

[10] Dillon W. P., Fishman R. A. (1998). Some lessons learned about the diagnosis and treatment of spontaneous intracranial hypotension. *American Journal of Neuroradiology*.

[11] Goadsby P. J., Boes C. (2002). New daily persistent headache. *Journal of Neurology, Neurosurgery & Psychiatry*.

[12] Burtis M. T., Ulmer J. L., Miller G. A., Barboli A. C., Koss S. A., Brown W. D. (2005). Intradural spinal vein enlargement in craniospinal hypotension. *American Journal of Neuroradiology*.

[13] Inamasu J., Guiot B. H. (2006). Intracranial hypotension with spinal pathology. *Spine Journal*.

[14] Ropper A. H., Brown R. H., Ropper A. H., Brown R. H. (2005). Disease of the peripheral nerves. *Adams and Victor's Principles of Neurology*.

[15] Gilden D. H., Wright R. R., Schneck S. A., Gwaltney J. M., Mahalingam R. (1994). Zoster sine herpete, a clinical variant. *Annals of Neurology*.

[16] Mayo D. R., Booss J. (1989). Varicella zoster—associated neurologic disease without skin lesions. *Archives of Neurology*.

[17] Mokri B., Atkinson J. L. D., Piepgras D. G. (2000). Absent headache despite CSF volume depletion (intracranial hypotension). *Neurology*.

[18] Hochman M. S., Naidich T. P. (1999). Diffuse meningeal enhancement in patients with overdraining, long-standing ventricular shunts. *Neurology*.

[19] Schievink W. I., Tourje J. (2000). Intracranial hypotension without meningeal enhancement on magnetic resonance imaging: case report. *Journal of Neurosurgery*.

[20] Schoffer K. L., Benstead T. J., Grant I. (2002). Spontaneous intracranial hypotension in the absence of magnetic resonance imaging abnormalities. *Canadian Journal of Neurological Sciences*.

[21] Rando T. A., Fishman R. A. (1992). Spontaneous intracranial hypotension: report of two cases and review of the literature. *Neurology*.

[22] Chen C.-J., Lee T.-H., Hsu H.-L., Tseung Y.-C., Wong Y.-C., Wang L.-J. (2002). Spinal MR findings in spontaneous intracranial hypotension. *Neuroradiology*.

[23] Chiapparini L., Farina L., D'Incerti L. (2002). Spinal radiological findings in nine patients with spontaneous intracranial hypotension. *Neuroradiology*.

[24] Moayeri N. N., Henson J. W., Schaefer P. W., Zervas N. T. (1998). Spinal dural enhancement on magnetic resonance imaging associated with spontaneous intracranial hypotension: report of three cases and review of the literature. *Journal of Neurosurgery*.

[25] Rabin B. M., Roychowdhury S., Meyer J. R., Cohen B. A., LaPat K. D., Russell E. J. (1998). Spontaneous intracranial hypotension: spinal MR findings. *American Journal of Neuroradiology*.

